# End-of-Life Symptom Burden among Patients with Cancer Who Were Provided Medical Assistance in Dying (MAID): A Longitudinal Propensity-Score-Matched Cohort Study

**DOI:** 10.3390/cancers16071294

**Published:** 2024-03-27

**Authors:** K. Brooke Russell, Caitlin Forbes, Siwei Qi, Claire Link, Linda Watson, Andrea Deiure, Shuang Lu, James Silvius, Brian Kelly, Barry D. Bultz, Fiona Schulte

**Affiliations:** 1 Department of Psychology, University of Calgary, Calgary, AB T2N 1N4, Canada; kbrussel@ucalgary.ca; 2 Department of Oncology, Division of Psychosocial Oncology, University of Calgary, Calgary, AB T2N 1N4, Canada; caitlin.forbes@ucalgary.ca (C.F.); brian.kelly@newcastle.edu.au (B.K.); bdbultz@ucalgary.ca (B.D.B.); 3 Applied Research and Patient Experience, Cancer Care Alberta, Alberta Health Services, Calgary, AB T2S 3C1, Canada; siwei.qi@albertahealthservices.ca (S.Q.); claire.link@albertahealthservices.ca (C.L.); linda.watson@albertahealthservices.ca (L.W.); andrea.deiure@albertahealthservices.ca (A.D.); 4 Faculty of Nursing, University of Calgary, Calgary, AB T2N 1N4, Canada; 5 Surveillance and Reporting, Cancer Care Alberta, Alberta Health Services, Calgary, AB T2S 3C3, Canada; shuang.lu3@iqvia.com; 6 Cumming School of Medicine, University of Calgary, Calgary, AB T2N 1N4, Canada; james.silvius@albertahealthservices.ca; 7 Provincial Seniors Health and Continuing Care, Alberta Health Services, Calgary, Alberta T2W 1S7, Canada; 8 School of Medicine and Public Health, University of Newcastle, Callaghan, NSW 2308, Australia

**Keywords:** patient-reported outcomes, symptom burden, palliative care, end-of-life care, medical assistance in dying (MAID)

## Abstract

**Simple Summary:**

Cancer is the most common medical condition among Canadians who receive Medical Assistance in Dying (MAID). We aimed to evaluate if people who choose MAID have worse symptoms compared to those who do not. Using data from cancer patients in Alberta who died between July 2017 and January 2019, we carefully compared two groups: those who received MAID (*n* = 149) and those who did not receive MAID (*n* = 149). Both groups had worsening symptoms in the year before death. Those who received MAID had slightly worse anxiety and slightly worse lack of appetite than those who did not receive MAID. Overall, our results showed that symptoms between the groups were similar, and that those who received MAID did not have worse overall symptoms. This study highlights the importance of regular symptom screening and early symptom intervention for all patients with advancing cancer.

**Abstract:**

Cancer is the primary underlying condition for most Canadians who are provided Medical Assistance in Dying (MAID). However, it is unknown whether cancer patients who are provided MAID experience disproportionally higher symptom burden compared to those who are not provided MAID. Thus, we used a propensity-score-matched cohort design to evaluate longitudinal symptom trajectories over the last 12 months of patients’ lives, comparing cancer patients in Alberta who were and were not provided MAID. We utilized routinely collected retrospective Patient-Reported Outcomes (PROs) data from the Edmonton Symptom Assessment System (ESAS-r) reported by Albertans with cancer who died between July 2017 and January 2019. The data were analyzed using mixed-effect models for repeated measures to compare differences in symptom trajectories between the cohorts over time. Both cohorts experienced increasing severity in all symptoms in the year prior to death (β from 0.086 to 0.231, *p* ≤ .001 to .002). Those in the MAID cohort reported significantly greater anxiety (β = −0.831, *p* = .044) and greater lack of appetite (β = −0.934, *p* = .039) compared to those in the non-MAID cohort. The majority (65.8%) of patients who received MAID submitted their request for MAID within one month of their death. Overall, the MAID patients did not experience disproportionally higher symptom burden. These results emphasize opportunities to address patient suffering for all patients with cancer through routine collection of PROs as well as targeted and early palliative approaches to care.

## 1. Introduction

Patients with cancer can experience significant suffering due to both physical (e.g., pain, fatigue, shortness of breath) and psychological symptoms (e.g., anxiety, depression) [1,2,3,4]. Given the heterogeneity of cancer diagnoses, treatments, and individual factors such as comorbidities, the prevalence, type, and severity of symptoms vary widely among patients and over time [5,6,7]. Patients with cancer often experience a complex burden of multiple co-occurring symptoms [8,9]. While symptom burden may be present at any point in a patient’s cancer trajectory, these symptoms are usually experienced with a greater frequency and intensity as death approaches [10,11]. Not surprisingly, it is well established that greater symptom burden is associated with a poorer quality of life [12,13,14,15]. In recent years, there has been a push for the integration of early palliative care in oncology settings, particularly for patients at greatest risk of suffering [16]. This targeted and early approach can improve patient wellbeing and act as a catalyst for conversations about goals of care and treatment planning [17]. Nevertheless, death remains inevitable for many patients with an advancing disease.

In recent years, legislation has been introduced to permit active interventions to deliberately hasten death for patients with advancing illnesses. Canada introduced Medical Assistance in Dying (MAID) legislation in 2016, which decriminalized medically assisted death across the country [18]. To access MAID, individuals must be at least 18 years of age, have the capacity to make their own healthcare decisions, and be experiencing a grievous or irremediable medical condition. At the time of this study, the criteria also state that patients must be in an irreversible advanced state of decline and be experiencing unbearable, untreatable suffering [19]. Consistent with other jurisdictions around the globe, cancer is the most frequently cited underlying medical condition for those who are provided MAID in Canada (65.6% in 2021) [20].

Recent evidence suggests that patients with cancer who are provided MAID report significant increases in symptom burden in the last year of their life [21]. However, it is not known how this increasing pattern of symptoms may be similar or different from those with cancer who die without the provision of MAID. Developing an understanding of whether patients who are provided MAID experience disproportionally higher symptom burden compared to those who do not request MAID is needed in order to identify potential unmet needs of this group, and clinically, could inform the identification of patients who would benefit from earlier interventions [16]. Thus, the aim of the present study was to compare the symptom characteristics and trajectories between patients with cancer who were provided MAID and patients with cancer who died without the provision of MAID, in the province of Alberta, Canada.

## 2. Materials and Methods

### 2.1. Study Design

To conduct this study, we followed STROBE guidelines for reporting observational studies [22]. This retrospective cohort study utilized administrative data from Alberta’s provincial MAID registry, Cancer Care Alberta’s (the provincial cancer program) electronic medical record, the Alberta Cancer Registry, and Patient-Reported Outcomes (PROs) data collected through chart audits. This study involved confidential patient data and was approved by the Health Research Ethics Board of Alberta—Cancer Committee (HREBA.CC-18-0709).

### 2.2. Propensity Score Matching

Propensity score matching (PSM) has been widely used to reduce confounding biases in observational studies and is one of the most useful tools for such studies [23]. A propensity score was estimated for each subject using a logistic regression model with all demographic and clinical characteristics (described below) that were available at the first time point (12 months before death) [24,25]. Nearest-neighbor matching was performed to match patients in the MAID and non-MAID cohorts (defined below), based on the estimated propensity scores [24]. If multiple subjects in the non-MAID cohort had propensity scores that were equally similar (the same absolute difference) to a subject in the MAID cohort, a subject in the non-MAID cohort was selected at random from those with the same score [24].

### 2.3. Study Sample

**Figure 1:**
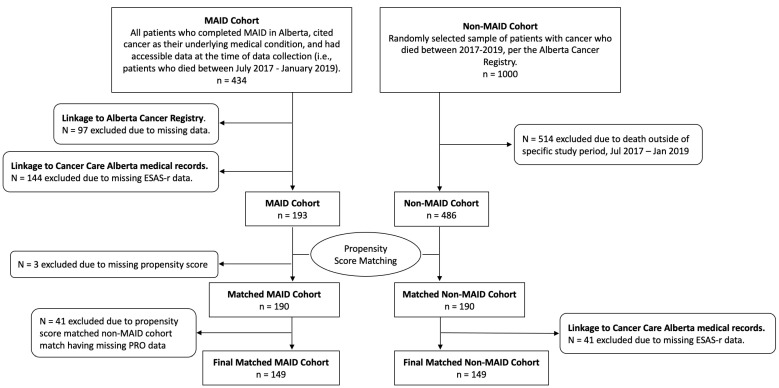
Patient flow diagram.

The study sample was composed of two patient cohorts (see Figure 1 for a detailed flow chart of the sample determination). The first cohort (“MAID cohort”) consisted of cancer patients who were provided MAID in Alberta between July 2017 and January 2019 (identified through the provincial MAID registry) and had completed at least one PROs questionnaire in the last 12 months of their life. In total, 337 patients were provided MAID in this time period. Of these patients, 188 were excluded for either not having PROs data (*n* = 144) or not having a propensity score due to missing data (*n* = 3), or due to their propensity-score-matched non-MAID cohort counterpart having no PROs data (*n* = 41). As a result, 149 patients were included in the final MAID cohort.

For comparison, we selected a cohort of cancer patients who died without the provision of MAID in Alberta (“non-MAID” cohort) during the same period (between July 2017 and January 2019) and who had completed at least one PROs questionnaire in the last 12 months of their life. From the population of all patients with cancer who died without the provision of MAID in Alberta between 2017 and 2019, Cancer Care Alberta’s Surveillance and Reporting data department randomly selected 1000 cancer patients and provided the study team with their available Alberta Cancer Registry data. To ensure we were capturing potential seasonal effects of symptom burden, our team further selected patients who died within the same months as the MAID cohort (July 2017 and January 2019) (*n* = 486). We then matched the MAID and non-MAID cohorts using PSM. Following this, 41 patients were excluded for not having PROs data. PROs data for the non-MAID cohort were not obtained until after the PSM was completed, due to resource limitations.

### 2.4. Measures

The PROs questionnaire used throughout Cancer Care Alberta includes the Edmonton Symptom Assessment System—Revised (ESAS-r), a self-report questionnaire measuring nine symptoms: pain, tiredness, drowsiness, nausea, lack of appetite, shortness of breath, depression, anxiety, and wellbeing [26]. Patients rate each symptom from 0 to 10, with 10 indicating the highest severity. This questionnaire was first developed for use with palliative cancer patients and has been used extensively in Alberta cancer centers due to its strong psychometric properties [27]. Patients seen in Cancer Care Alberta are routinely asked to complete this PROs questionnaire at clinic appointments as multifactorial Screening for Distress is an accreditation standard and requirement for cancer care in Canada [28]. Across all patient appointments that occur within Cancer Care Alberta, the completion rate for the PROs questionnaire is around 75% [29].

Demographic variables routinely collected at the time of enrollment in the cancer registry were used in the PSM and included the following: age at diagnosis; sex; marital status; and country of birth. Marital status classified patients as “Partnered” (i.e., married or in common-law relationships) or “Not Partnered” (i.e., single, divorced, or widowed). Rurality [30] and the neighborhood-level income quintile were also included in the PSM. Rurality was determined by using a patient’s most recent postal code and an Alberta-specific index [30]. Patients were assigned to one of three groups: (1) “Metro” (i.e., metropolitan or metropolitan-influenced area), (2) “Urban” (i.e., urban or urban-influenced area), and (3) “Rural” (i.e., large rural center and surrounding area, rural area, or remote area). The neighborhood-level income quintile was also based on the patients’ most recent postal codes. Country of birth classified patients into one of two groups: (1) Canada; (2) Outside of Canada. Finally, age at death was also recorded in the provincial registry and used in the present study.

The clinical variables retrieved from Cancer Care Alberta’s electronic medical record included the patient’s tumor type and grouping, and the number of comorbidities, coded using the Charlson Comorbidity Index [31,32,33]. We used a modified version of the Charlson Comorbidity Index, which excluded cancer and associated metastases as contributing factors, as all patients had a cancer diagnosis. These variables were also included in the PSM.

### 2.5. Statistical Analyses

Descriptive statistics (mean, standard deviation, frequency, and percentage) were used to report demographic and clinical characteristics for each cohort. Chi-squared tests and independent *t*-tests were used to examine the differences in these characteristics, as appropriate. The main analysis utilized mixed-effect models for repeated measures of symptom scores to test symptom differences between the cohorts over time. The independent variables in the mixed-effect model included time to death in months, cohort (MAID or non-MAID), and an interaction between the cohort and time as fixed effects.

Each model employed an unstructured covariance structure for random effects to address within-subject correlations [34]. Nine mixed-effect models were constructed in total, one for each ESAS-r symptom (used as the dependent variable). ESAS-r symptom scores from PROs questionnaires were ordered from 12 months before death (“12”) to 1 month before death (“1”). When more than one PRO questionnaire was completed by a patient in a single month, the questionnaire with higher symptom scores was selected, as is consistent with published research using similar methods [21,35]. Data were assumed to be missing at random, as is common when using real-world mortality data [36]. Statistical significance was set at *p* < .05, and the data were analyzed using SPSS Version 25.0 (Chicago, IL, USA) and R Version 3.6.2 (R Foundation for Statistical Computing) for PSM.

## 3. Results

### 3.1. Sample Characteristics

Table 1 presents a summary of the study sample according to the demographic and clinical characteristics of each cohort before and after PSM was performed. Before PSM, the MAID and non-MAID cohorts significantly differed on six out of nine characteristics: age at death (*p* < .01), rurality (*p* < .01), neighborhood-level income quintile (*p* < .01), tumor group (*p* < .01), age at diagnosis (*p* < .01), and the Charlson Comorbidity Index (*p* < .01).

All significant differences in characteristics between the cohorts were eliminated after matching for all nine characteristics using PSM (*p*-values = .163 to .948). The matched pairs were used in subsequent analyses for direct comparison, without further controlling the confounding effects of demographic and clinical characteristics [37,38,39,40].

Finally, the majority of patients (65.8%) in the MAID cohort submitted requests for MAID within the same month as their death (range ≥ 10 months–1 month).

### 3.2. Symptom Trajectories

**Figure 2:**
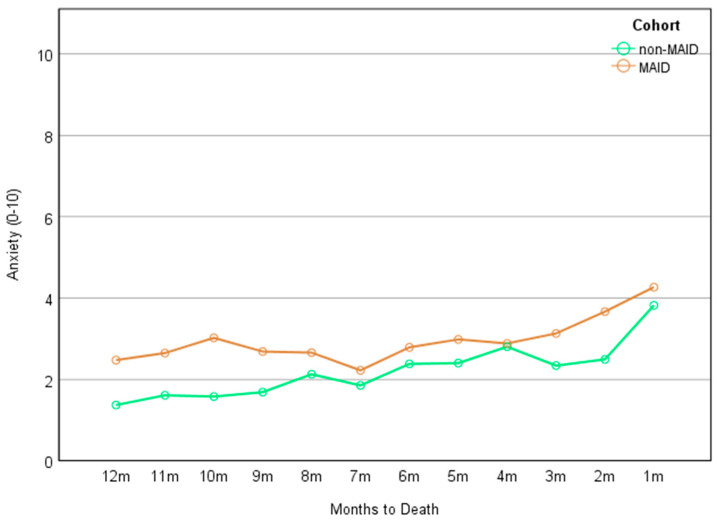
Anxiety across the months by cohort.

**Figure 3:**
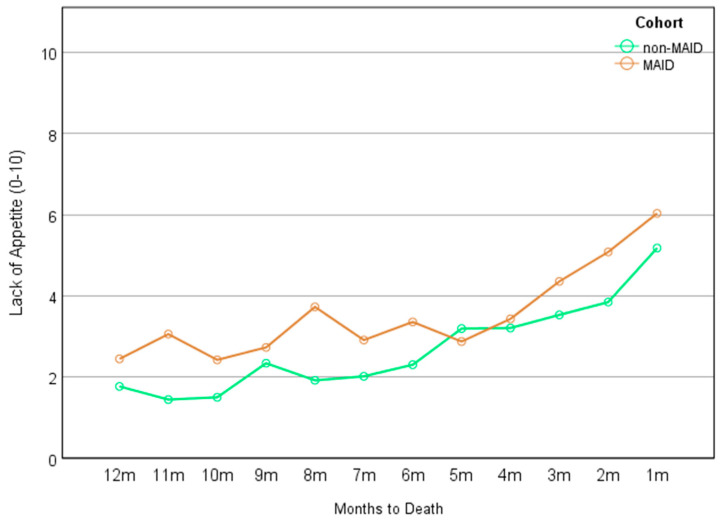
Lack of appetite across the months by cohort.

Detailed results of the mixed-effect models for each symptom can be found in Table 2. A significant main effect of time was found for each of the nine ESAS-r symptoms (*β* ranging from 0.086 to 0.231, *p* ranging from .002 to < .001), indicating that each symptom increased over time for both cohorts. In addition, there was a significant main effect of group on symptoms of anxiety (*β* = −0.831, *p* = .044) and lack of appetite (*β* = −0.934, *p* = .039), indicating that statistically, the MAID cohort experienced significantly greater anxiety (Figure 2) and greater lack of appetite (Figure 3) than the non-MAID cohort across the 12-month time frame. These differences neared an estimated clinically significant one-point difference in scores [41,42]. No significant cohort × time interactions were noted for any of the nine ESAS-r symptoms.

## 4. Discussion

Given the increasing use of MAID in Canada and internationally, there is a growing need to better understand the symptom-related experiences of patients with cancer who choose to receive MAID [20,43] in order to identify potential unmet needs and ensure appropriate and effective symptom management in the months leading to death. Using routinely gathered PROs data, our retrospective, real-world study aimed to examine longitudinal symptom burden trajectories in the last 12 months of life among patients with cancer who requested and were ultimately provided MAID in comparison to a propensity-score-matched cohort of those with cancer who died without the provision of MAID. Consistent with previous reports in the literature, our findings revealed that patients in both cohorts reported increasing severity of symptoms in the months leading to their death. Our data reflect that overall, patients who were provided MAID may have experienced slightly higher symptom burden than those who died without MAID, though these findings were modest. Specifically, we observed that those who were ultimately provided MAID reported statistically greater lack of appetite and greater anxiety symptoms across the last 12 months of their life in comparison to their matched non-MAID counterparts. These differences approached clinical significance.

Previous studies evaluating the wish to hasten death for those with advancing cancer have been primarily cross-sectional in nature and have more frequently explored the relationship between depression and the theoretical wish to hasten death. However, our longitudinal real-world retrospective clinical data highlight anxiety symptoms as an important area for clinical attention. Evidence from caregivers suggests that patients with advancing cancer worry about the loss of cognitive capacity resulting in an inability to engage meaningfully with their loved ones (e.g., to say goodbye), loss of physical capacity resulting in dependency on others, and loss of dignity [44]. Patients may also worry about being a burden to others, reflected by evidence that those with better-perceived social support experience more anxiety symptoms at the end of their life and worry about how their death will impact their loved ones [45,46]. Indeed, intolerance of uncertainty is a key factor in anxiety and, to a lesser extent, depression [47]. Given the deep uncertainty that is inherent with cancer from diagnosis to cancer-related death, it is possible that patients with greater anxiety throughout their cancer trajectory desired increased certainty over the circumstances of their death, potentially influencing their interest in MAID. Importantly, our results suggest that differences in anxiety levels between the two cohorts existed even prior to formal requests for MAID had been submitted, as the majority of requests for MAID occurred within one month of death.

Our results also indicate that those who were ultimately provided MAID had a greater lack of appetite in the months leading to their death than their non-MAID counterparts. While lack of appetite is not uncommon for patients with cancer, over time, lack of appetite can produce significant challenges that interfere with daily life such as weakness and reduced energy, leading to increased dependence on others, psychosocial distress, and loss of opportunities for social connection [48,49]. Anxiety may also impact appetite loss, with nausea and gastrointestinal symptoms being common features of anxiety [50]—the interaction of these symptoms is an important area for further investigation. In this way, greater lack of appetite experienced throughout the cancer trajectory may be associated with greater suffering and thus an increased desire to hasten death via MAID. These findings are in contrast to those of a similar investigation conducted in the Netherlands by Ruijs, which examined end-of-life symptom burden in patients with advanced cancer who did and did not request assisted death [51]. In their study, Ruijs [51] found no significant differences in symptom-related unbearable suffering between the two groups, including lack of appetite and anxiety. Importantly, the methodology of our study was quite different, including the use of longitudinal vs. cross-sectional data, matched vs. unmatched groups, continuous vs. dichotomized data, a focus on symptom intensity vs. unbearable suffering, and a larger, balanced sample size. Considering these methodological disparities, it is possible that the design of our study was more robust to detect subtle differences between groups, as reflected in our observed findings.

Importantly, our findings highlight that both cohorts exhibited high and increasing symptom burden across the 12 months prior to their death. These findings emphasize the utility of PROs to tailor symptom management to the unique needs of each patient and highlight the potential importance of anxiety and lack of appetite, consistent with previous recommendations [52]. PROs can also lead to patient-driven conversations about symptoms, palliative care, and end-of-life care instead of relying on clinicians to initiate these conversations. Systematic implementation of Screening for Distress and the integration of psychosocial care into cancer services has become a global priority in cancer policy and has demonstrated an ability to improve clinical outcomes [53]. However, challenges remain in building and sustaining the necessary models of care to support these goals [54]. Although challenges in integrating psychosocial care exist at the individual, intervention, and organizational levels, stepped-care approaches and the collection of online, community, and self-help resources may extend referral options and work to address gaps in care [55]. Psychosocial and palliative aspects of care must include the ability for the early identification of patients at greatest risk to increase the intensity of interventions for persistent symptoms through prompt assessment, and linkage to appropriate care and support.

These findings contribute new knowledge by identifying differences in symptom trajectories between matched cohorts of cancer patients who were and were not provided MAID, and using data from real-world routine PROs to better understand the clinical factors associated with cancer patients who are provided MAID. Previous cross-sectional research has identified a trend of increasing physical symptom burden closer to the time of death [10,11,56]. However, there has not been any evidence reported regarding changes in psychological symptoms over time [10,11,56]. The present study differs in its use of longitudinal data over a 12-month prior-to-death period, and therefore had the capacity to detect within-cohort changes over a longer period of time. This work indicates that both cohorts experienced an increase in their physical and psychosocial symptom burden as they approached death, emphasizing the importance of symptom management.

This work is limited by the fact that it is an observational decedent cohort study, which may have confounded our results [57]. Nevertheless, being unable to conduct a randomized control trial (RCT) in this context, this study offers important insights into the trajectory of symptoms experienced by these two cohorts [58,59]. With respect to generalizability, our cohorts were broadly reflective of the broader population of Albertans with cancer with respect to sex, marital status, and country of birth. However, given that we matched our non-MAID cohort to our MAID cohort, our pre-matching comparison of groups suggests that our sample may overrepresent slightly younger patients in Metro areas, those with no comorbidities, and those with higher incomes. Our data also suggest that patients within gynecological cancers may have been overrepresented, while those with lung cancer may have been underrepresented. Furthermore, we were limited by the use of available registry data in Alberta. Variables such as ethnicity, religion, and access to palliative care services were not available to add further context to the decision to pursue MAID, or to include in the propensity score matching [58]. Mixed-effect models were used to manage the large amount of missing data; this is an effective method, but there is a risk of bias if data are not missing at random as is assumed [34]. Finally, we cannot be sure that all patients in the non-MAID cohort passed away as a result of their advancing cancer; it is possible that some patients passed away from other conditions or circumstances.

## 5. Conclusions

Overall, our results suggest that Albertan cancer patients who were provided MAID experienced modestly higher symptom burden compared to those who were not provided MAID. Specifically, we observed that patients who received MAID reported higher overall levels of anxiety and appetite loss in the 12 months prior to their death compared to those who died without the provision of MAID. These results point to specific symptoms which can be identified in clinical settings and highlight foci for clinical intervention. This is the first study that has combined real-world clinical data, including routinely collected PROs, and the provision of MAID using a matched-cohort design. It also highlights the value of examining routinely collected PROs for important clinical issues that can be applied to both research and clinical practice related to end-of-life care. Future longitudinal studies and qualitative exploration of these symptoms may assist in helping to better characterize and understand the experiences and sources of anxiety and lack of appetite for cancer patients considering MAID. It is critical that we develop a deeper understanding of how symptoms may play a role in patients’ wishes to be provided MAID in order to improve the overall quality of care and quality of life of these patients.

## Figures and Tables

**Table 1:** Demographic and clinical characteristics before and after PSM.

	Before PSM			After PSM		
	MAID Cohort (*n* = 149) ^a^	Full Non-MAID Cohort (*n* = 486) ^a^	*p*	MAID Cohort (*n* = 149) ^a^	Matched Non-MAID Cohort (*n* = 149) ^a^	*p*
Age at death [mean (SD)]	68.7 (11.8)	72.5 (12.6)	.001	68.7 (11.8)	67.4 (11.2)	.346
Sex:			.499			.163
Female	74 (49.7%)	226 (46.5%)		74 (49.7%)	62 (41.6%)	
Male	75 (50.3%)	260 (53.5%)		75 (50.3%)	87 (58.4%)	
Marital status:			.677			.557
Married/in common-law relationship	84 (56.4%)	227 (58.4%)		84 (56.4%)	89 (59.7%)	
Single/widowed/divorced/separated	65 (43.6%)	162 (41.6%)		65 (43.6%)	60 (40.3%)	
Rurality:			.000			.712
Metro	113 (75.8%)	282 (58.1%)		113 (75.8%)	112 (75.2%)	
Urban	16 (10.7%)	66 (13.6%)		16 (10.7%)	13 (8.7%)	
Rural	20 (13.4%)	137 (28.2%)		20 (13.4%)	24 (16.1%)	
Neighborhood-level income quintile:			.005			.461
1 (USD 19,968–USD 62,933) (lowest)	22 (14.8%)	100 (20.7%)		22 (14.8%)	30 (20.1%)	
2 (USD 63,040–USD 77,248)	25 (16.8%)	112 (23.2%)		25 (16.8%)	31 (20.8%)	
3 (USD 77,312–USD 96,000)	23 (15.4%)	94 (19.5%)		23 (15.4%)	17 (11.4%)	
4 (USD 96,256–USD 120,064)	33 (22.1%)	91 (18.8%)		33 (22.1%)	26 (17.4%)	
5 (USD 120,192–USD 312,320) (highest)	46 (30.9%)	86 (17.8%)		46 (30.9%)	45 (30.2%)	
Birth country:			.208			.878
Canada	125 (83.9%)	306 (79.1%)		125 (83.9%)	124 (83.2%)	
Outside of Canada (high income)	24 (16.1%)	81 (20.9%)		24 (16.1%)	25 (16.8%)	
Tumor group:			.003			.899
Breast	13 (8.7%)	46 (9.5%)		13 (8.7%)	13 (8.7%)	
Gastrointestinal	44 (29.5%)	150 (30.9%)		44 (29.5%)	43 (28.9%)	
Genitourinary	14 (9.4%)	55 (11.3%)		14 (9.4%)	21 (14.1%)	
Gynecology	17 (11.4%)	24 (4.9%)		17 (11.4%)	14 (9.4%)	
Hematology	15 (10.1%)	51 (10.5%)		15 (10.1%)	12 (8.1%)	
Lung	23 (15.4%)	122 (25.1%)		23 (15.4%)	25 (16.8%)	
Other ^b^	23 (15.4%)	38 (7.8%)		23 (15.4%)	21 (14.1%)	
Age at diagnosis:			.000			.948
Mean (SD)	64.0 (12.3)	69.2 (13.1)		64.0 (12.3)	64.1 (10.8)	
CCI:			.000			.900
0	104 (69.8%)	207 (42.6%)		104 (69.8%)	103 (69.1%)	
≥1	45 (30.2%)	279 (57.4%)		45 (30.2%)	46 (30.9%)	

^a^ The sample size for each variable may differ from the total cohort size, due to some patients having missing data for certain characteristics. ^b^ Other includes Central Nervous System, endocrine, head-and-neck, melanoma, non-melanoma skin, sarcoma, and other malignant cancers.

**Table 2:** Summary of the MEM results.

	Cohort ^a^		Time ^b^		Interaction	
	β (95% CI)	*p*	β (95% CI)	*p*	β (95% CI)	*p*
Pain	−0.305 (−1.104–0.493)	.453	0.223 (0.161–0.287)	.000	−0.016 (−0.100–0.068)	.709
Tiredness	−0.211 (−1.001–0.586)	.602	0.231 (0.169–0.294)	.000	−0.038 (−0.123–0.046)	.372
Drowsiness	−0.381 (−1.185–0.424)	.353	0.219 (0.156–0.281)	.000	−0.076 (−0.160–0.008)	.077
Nausea	−0.172 (−0.859–0.516)	.624	0.175 (0.119–0.231)	.000	−0.064 (−0.139–0.012)	.101
Lack of appetite	−0.934 (−1.823–−0.016)	.039	0.230 (0.159–0.301)	.000	0.046 (−0.050–0.141)	.348
Shortness of breath	−0.379 (−1.174–0.417)	.350	0.171 (0.112–0.230)	.000	−0.002 (−0.081–0.078)	.969
Depression	−0.106 (−0.902–0.688)	.792	0.164 (0.111–0.217)	.000	−0.039 (−0.110–0.031)	.275
Anxiety	−0.831 (−1.641–−0.022)	.044	0.086 (0.030–0.141)	.002	0.044 (−0.030–0.119)	.246
Wellbeing	−0.107 (−0.888–0.675)	.789	0.231 (0.170–0.291)	.000	−0.043 (−0.125–0.039)	.308

^a^ The model employs the MAID cohort as a reference, and β represents the parameter estimate (i.e., estimated difference in average numeric rating scale) for the non-MAID cohort relative to the MAID cohort. Negative values of β indicate lower scores in the non-MAID cohort compared to the MAID cohort. ^b^ The model treats time as a continuous variable. Positive values of the β parameter suggest that the symptom in question escalated as time approached the provision of MAID. Note: Not all patients completed questionnaires at each time point. Details regarding the missing data are available upon request.

## Data Availability

The raw data supporting the conclusions of this article will be made available by the authors on reasonable request.
